# Sexual Interactions Influence the Molecular Oscillations in DN1 Pacemaker Neurons in *Drosophila melanogaster*

**DOI:** 10.1371/journal.pone.0084495

**Published:** 2013-12-18

**Authors:** Shiho Hanafusa, Tomoaki Kawaguchi, Yujiro Umezaki, Kenji Tomioka, Taishi Yoshii

**Affiliations:** Graduate School of Natural Science and Technology, Okayama University, Okayama, Japan; University of Massachusetts Medical School, United States of America

## Abstract

Circadian rhythms can synchronize to environmental time cues, such as light, temperature, humidity, and food availability. Previous studies have suggested that these rhythms can also be entrained by social interactions. Here, we used *Drosophila melanogaster* as a model to study the influence of socio-sexual interactions on the circadian clock in behavior and pacemaker neurons. If two flies of opposite sex were paired and kept in a small space, the daily activity patterns of the two flies were clearly different from the sum of the activity of single male and female flies. Compared with single flies, paired flies were more active in the night and morning, were more active during females’ active phase, and were less active during males’ active phase. These behavioral phenotypes are related to courtship behavior, but not to the circadian clock. Nevertheless, in male-female pairs of flies with clocks at different speeds (wild-type and *per* ^*S*^ flies), clock protein cycling in the DN1 pacemaker neurons in the male brain were slightly influenced by their partners. These results suggest that sexual interactions between male-female couples can serve as a weak zeitgeber for the DN1 pacemaker neurons, but the effect is not sufficient to alter rhythms of behavioral activity.

## Introduction

 The circadian clock synchronizes with several environmental stimuli in order to precisely predict 24-h environmental changes. Daily light and temperature cycles are the most powerful time-givers—zeitgebers—for the clock and they are directly generated by the Earth’s rotation. Other environmental factors that are not directly generated by the Earth’s rotation, but are generated by consequences of circadian rhythms in ecological systems, are often discussed as potential zeitgebers. One of these elusive factors is social interaction [1-3]. It would be advantageous for animals to predict the timing of any 24-h rhythms that might occur in, for instance, mutualism, parasitism, competition, or predator-prey interactions. Therefore, some animals may have evolved circadian clocks that use social stimuli as zeitgebers. 

 The honeybee is a good example of an animal that has circadian rhythms that are influenced by conspecific relationships. Worker bees, including young nurses taking care of the brood, are active during both day and night, and show no circadian rhythms. In contrast, older foragers have strong rhythms in foraging for nectar and pollen and visit flowers at a certain time of day [4]. Another solid example of social adaptation of circadian rhythms is maternal entrainment. In mammals (e.g., hamsters, mice, and rats), the circadian rhythms of mothers entrain the rhythms of their fetuses and pups until the young animals start to sense light-dark (LD) cycles [2]. In an insect model organism, *Drosophila melanogaster*, Levine and colleagues demonstrated that, when flies were kept in constant darkness (DD), individual flies kept in a group exhibited more coherent circadian phases than isolated flies [5]. Furthermore, phase coherence was stronger in larger groups.

 Due to the availability of outstanding genetic tools, *Drosophila* is one of the animals for which the study of the circadian clock is most advanced. The molecular mechanism of the clock has been unveiled by genetic screening and molecular biological techniques and is explained by the involvement of several genes that form autoregulatory feedback loops and cycle with a period of approximately 24 h [6,7]. These feedback loops generate molecular oscillations in the transcriptional and translational levels of the “clock” genes, including period (per), timeless (tim), *Clock* (*Clk*), *vrille* (*vri*), and *Pdp1-epsilon* (*Pdp1*). These molecular oscillations are analogous the minute-hand of a mechanical clock. 

Clock genes are expressed in ~150 neurons in the brain of *Drosophila*. These neurons control behavioral rhythms, and are therefore called pacemaker neurons [8]. These 150 neurons are divided into eight groups based on their location in the brain, the size of the cell, and neurotransmitter content. The DN1, DN2, and DN3 groups are in the posterior dorsal part of the brain, whereas the LNd, l-LNv, and s-LNv groups are in a relatively anterior and lateral part of the brain. The LPN group is located in the posterior lateral part of the brain. l-LNv and s-LNv neurons express a main circadian neurotransmitter, Pigment-dispersing factor (PDF) [9]. However, a single neuron among the s-LNv cells in each hemisphere does not express PDF and is called the 5^th^ s-LNv [10]. Each pacemaker neural group appears to play a distinct role in fly activity rhythms, which have one activity peak in the morning and another in the evening under LD cycles [11]. Some lines of evidence demonstrate that the s-LNv group is more important for the morning activity peak and the LNd group is more important for the evening peak [12,13]. Therefore, it is likely that the ~150 pacemaker neurons are functionally differentiated in order to coordinate the behavioral rhythms of flies.

 Despite the advanced studies of *Drosophila*, it is still not clear whether social interactions are used as a zeitgeber in these flies. This is because all of the studies to date have been at the behavioral level, but not at the level of pacemaker neurons. In *Drosophila*, sexual interaction has been extensively studied [14], and a previous study has shown that sexual interaction appears to influence circadian locomotor rhythms [15,16]. We attempted to clarify whether male-female interactions have any impact on the circadian clock. We used *per* ^*S*^ mutant flies that have a clock that is faster in DD (~19 h period) than that of wild-type (WT) files and display a phase-advanced evening activity peak in LD, to investigate whether the clock of WT flies can be influenced by the presence of *per* ^*S*^ flies of the opposite sex at behavioral and neural levels. Our study demonstrated that sexual interactions may serve as a weak zeitgeber in a specific pacemaker group in the brain of *Drosophila*. 

## Materials and Methods

### Fly strains

*Canton-S* flies were used as the wild-type flies. The clock mutants *per* ^01^ and *per* ^*S*^ have been described previously [17]. *fru* ^*F*^ flies express only a female specific splicing form of the fruitless (fru) gene, so that male *fru* ^*F*^ flies show very reduced courtship behavior to females [18]. *fru-gal4* [19], *per* ^01^ *w*;;*uas-per* [20], *w*;*Mai179-gal4* [12], and *y w*;;*Clk4.1M-gal4/TM6B* [21] were kindly donated by B.C. Dickson (Institute of Molecular Biotechnology of the Austrian Academy of Sciences), F. Rouyer (CNRS), and P. Emery (University of Massachusetts), respectively. *y w; uas-GFP.S65T* flies were obtained from the Bloomington Drosophila stock center. The flies were reared under LD 12:12 cycles on *Drosophila* medium (0.7% agar, 8.0% glucose, 3.3% yeast, 4.0% cornmeal, 2.5% wheat embryo, and 0.25% propionic acid) at 25°C. Only virgin male and female flies that were 3–6 days old were used for experiments.

### Activity recording

The activity of 3- to 6-day-old virgin male and virgin female flies was recorded, although male-female pairs immediately mated after they were placed together. The locomotor activity of flies was recorded using a conventional method in which a computer recorded the number of interruptions of an infrared beam in 6-min bins [22]. Individuals or pairs of flies were placed in rectangular acrylic tubes (3 x 3 x 70 mm) that contained fly food (1.5% agar and 8.0% glucose). The fly houses and activity monitors were placed in an incubator (MIR-153; Sanyo Biomedica, Osaka, Japan) in which light conditions were controlled using an electric timer. The light source was a 15-W cool white fluorescent lamp (FL15N; Panasonic, Tokyo, Japan). The light intensity was approximately 500 lux and the temperature was maintained constant at 25°C. LD cycles were composed of 12 h of light and 12 h of darkness (LD 12:12). Activity rhythms were recorded for 6 days and the data recorded on the first day was excluded from data analyses. For visual inspection, raw data were displayed as actograms using ActogramJ (http://actogramj.neurofly.de/) [23]. Six-min data were transformed to 30-min data and the daily activity profiles were averaged over a 5-day period (day 2-6) for individual flies or individual pairs. Then, the data were averaged across all flies or pairs of each group. 

### Immunohistochemistry

 Whole flies were fixed in 4% paraformaldehyde in phosphate-buffered saline (PBS) with 0.1% Triton X-100 for 2.5 h at room temperature (RT). The fixed flies were washed three times in PBS, and then the brains were dissected. Following washing three times with PBS containing 0.5% Triton-X (PBS-T), the brains were blocked in PBS-T containing 5% normal donkey serum for 2 h at RT, and subsequently incubated in primary antibodies at 4°C for 48 h. After washing six times in PBS-T, the brains were incubated with secondary antibodies at RT for 3 h. The brains were again washed six times in PBS-T and mounted in Vectashield mounting medium (Vector Laboratories, Burlingame, CA). Primary antibodies used here: mouse anti-GFP antibody (1:1,000)(Wako, Osaka, Japan); rabbit anti-Gryllus PDF antibody (1:6,000) [24]; rabbit anti-PDP1 antibody (1:3,000) [25]; and mouse anti-PDF antibody (1:1,000) (Developmental Studies Hybridoma Bank; [26]). Secondary antibodies used here included goat anti-mouse IgG Alexa488 (1:500) (Life technologies, Carlsbad, CA) and goat anti-rabbit IgG Cy3 (1:500) (Millipore, Billerica, MA). 

 Staining was visualized using a laser scanning confocal microscope (Fluoview300; Olympus, Tokyo, Japan). To quantify the number of clock neurons that express the *fru* gene, each single optical section of the confocal stack was analyzed. Cells, in which the *fru-gal4* driven GFP expression was colocalized with the clock neuron specific PDP1 antibody staining, were determined as *fru*-positive clock neurons. The number of *fru*-positive clock neurons was blindly scored for each sex. For quantification of immunostaining, the confocal microscope settings were maintained constant throughout the experiments. For each time-point, 10 hemispheres from 10 brains were analyzed. Measurement of staining intensity was performed using imageJ (http://rsb.info.nih.gov/ij/) as described previously [27]. For each time-point, a Kolmogorov-Smirnov test was used to determine whether the data were normally distributed. T-tests (for normally distributed data) and Mann-Whitney U-tests (for data not normally distributed) with Bonferroni correction were used to test for statistically significant differences between two groups at each time-point. These statistics were calculated using EZR software, which is based on R [28]. 

## Results

### Sexual interactions enhance nocturnality, but not via the molecular clock

**Figure 1:**
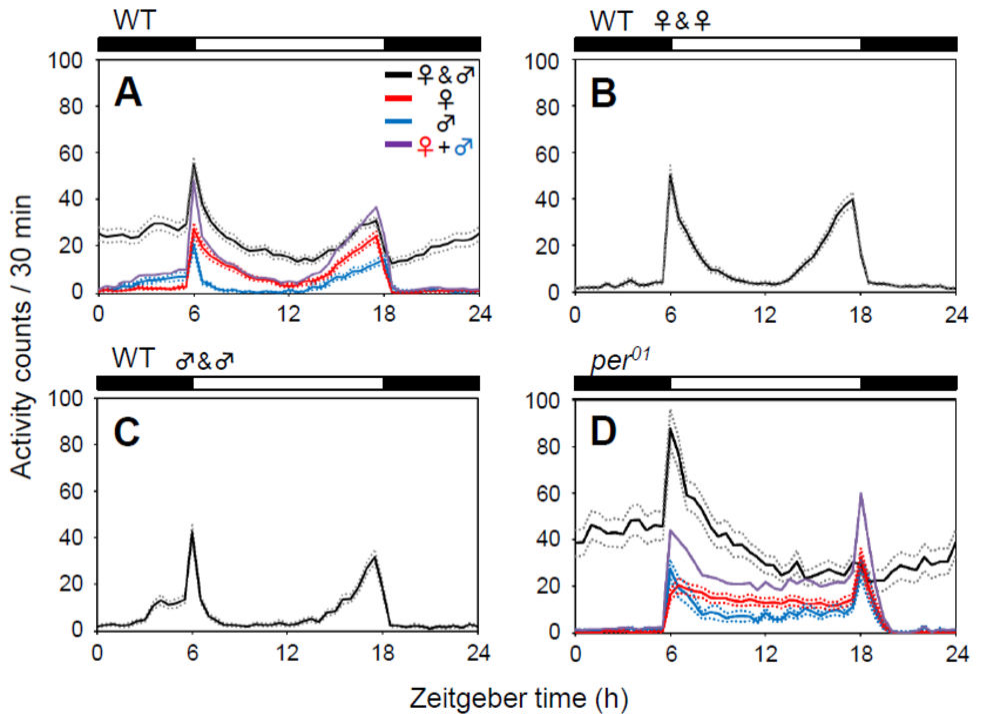
Effects of sexual interactions on the daily activity profiles. (A) Wild-type (WT) male and WT female *Drosophila* were paired in an activity recording tube, and the pooled activity of the two flies was recorded for 6 days. The average activity profile (± SEM) of the two flies (♀&♂) is plotted (A; black line, n = 122). SEM is shown by dotted lines. As controls, the average activity profiles of single male flies (♂, blue line, n = 39), of single female flies (♀, red line, n = 61), and the sum of the average activity of single male and single female flies (♀+♂, purple line) are also plotted on the same graph. Night activity was strongly enhanced in male-female pairs of flies. Activity profiles of female-female WT pairs (B), male-male WT pairs, (C) and *per* ^01^ mutant male-female pairs (♀&♂, black line, n = 42; ♂, blue line, n = 42; ♀, red line, n = 38; ♀+♂, purple line) (D). In *per* ^01^ male-female pairs, the morning activity increased as well as the night activity. Black or white bars above the graphs indicate light conditions.

 Because we used a conventional activity recording system, the activity of each male and female fly was pooled, as in a previous study [29]. Individual flies displayed typical bimodal activity patterns with peaks in the morning and evening (Figure 1A, red and blue, respectively). As reported previously [30], there is a sex-specific difference in daily activity patterns, as females were more active than males during the daytime. The pooled activity data for paired male and female flies indicates that they were more active at night than individually housed flies (Figure 1A). Such nocturnality was not observed in male-male pairs or female-female pairs (Figure 1B, C). This observation is consistent with those of previous studies [16,29]. To determine whether the nocturnality in male-female pairs is mediated by the circadian clock, we paired male and female *per* ^01^ arrhythmic mutants, in which the circadian feedback loops stop due to the lack of a functional *per* gene [31]. An increase of night activity and overall activity, relative to the activity of individually housed flies, was also observed for *per* ^01^ male-female pairs (Figure 1D). This suggests that the molecular clock is not involved in the observed increase in night activity. 

### The phase of the female’s evening activity influences the pooled activity profile

**Figure 2:**
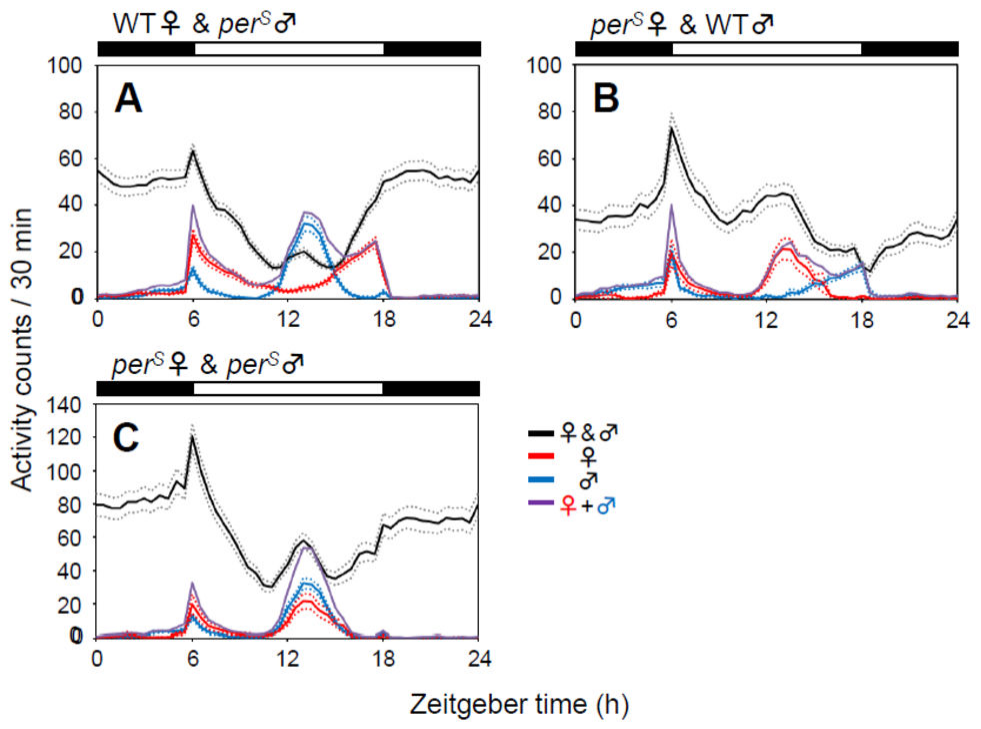
Activity profiles of pairs of WT and *per* ^*S*^ *Drosophila* flies. (A) WT females paired with perS males (black line, n = 151; blue line, n = 56; red line, n = 61). (B) perS females paired with WT males (black line, n = 75; blue line, n = 39; red line, n = 16). (C) perS females paired with perS males (black line, n = 73; blue line, n = 56; red line, n = 16). Activity of the two flies or single flies was recorded for 6 days in LD cycles and the average activity profiles were calculated from data of the last 5 days. In addition to high night and morning activity, activity corresponding to the females’ evening activity was enhanced, whereas the activity corresponding to the males’ evening activity was suppressed (A, B). For more detailed information, see Figure 1.

*per* ^*S*^ flies have an evening activity peak that is phase-advanced relative to that of WT (Figure 2A). Therefore, we paired *per* ^*S*^ and WT flies of opposite sex to determine whether there was any effect on activity rhythms. Surprisingly, for WT female-*per* ^*S*^ male pairs, the activity peak corresponding to the *per* ^*S*^ evening peak that occurs around zeitgeber time (ZT; ZT0 = lights-on, ZT12 = lights-off) 7 was suppressed, whereas the peak around lights-off that corresponds to the WT peak was strongly enhanced (Figure 2A). In *per* ^*S*^ female-WT male pairs, the evening peak corresponding to the WT peak was suppressed and the peak corresponding to *per* ^*S*^ flies was enhanced (Figure 2B). These data suggest that the phase of the females’ evening activity strongly influences the pooled activity profile. In both pairings, morning activity was also increased. Increased night and morning activity was observed for *per* ^*S*^ male- *per* ^*S*^ female pairs, as was observed for other pairs (Figure 2C). 

**Figure 3:**
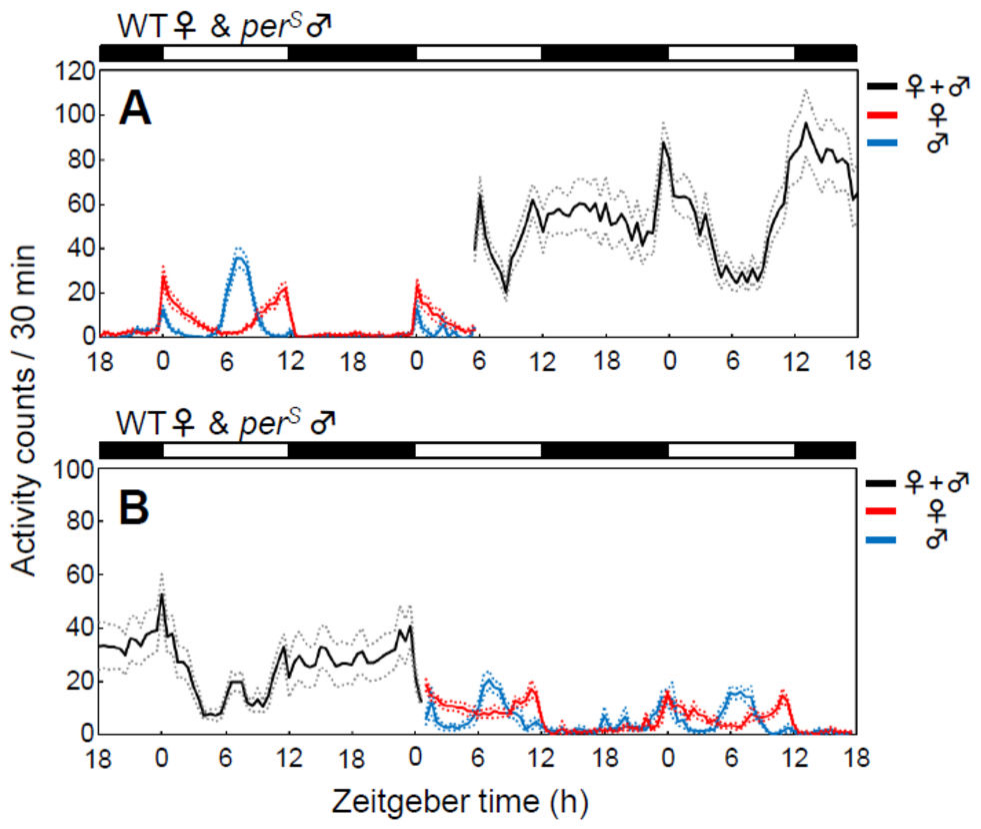
Time course analyses in pairing or splitting of mixed-sex *Drosophila* couples. After 6 days of recording, single WT females were either paired with *per* ^*S*^ male flies (A) (n = 45) or split from *per* ^*S*^ male flies (B) (n = 34). The average activity profiles of 3 consecutive days are shown. In both cases, activity patterns changed immediately to the new patterns (those of paired flies or those of single flies). For more detailed information, see Figure 1.

 Because the circadian clock usually needs several cycles to adapt to a new phase of zeitgeber [32,33], we observed the activity patterns before and after pairing male and female flies. Immediately after a WT female was paired with a *per* ^*S*^ male at ZT 6, activity increased at night and the male’s evening activity was suppressed without any indication of slow circadian adaptation (Figure 3A). The same was true for WT females-*per* ^*S*^ male pairs which were separated at ZT 0 (Figure 3B); the *per* ^*S*^ evening peak immediately reappeared. Given the fact, that the clock of *Drosophila* needs at least one entire day to re-synchronize to a new phase of LD cycle, even with light being the strongest zeitgeber [33,34], it would be unlikely that the male-female interaction can synchronize the clock much faster than light and that the clock is immediately reset by isolation of the two flies within few hours. Therefore, the activity modulation in male-female pairs may not be due to circadian entrainment between female and male flies. In WT female-*per* ^*S*^ male pairs in Figure 3B an early peak associated with *per* ^*S*^ males’ evening peak was visible at the middle of day, already before the isolation of the two flies. However, the peak was not very strong. This suggests that the male’s evening peak may be hidden behind overall high activity.

### The activity modulation induced by heterosexual couplings is suppressed in fru^F^ mutants

**Figure 4:**
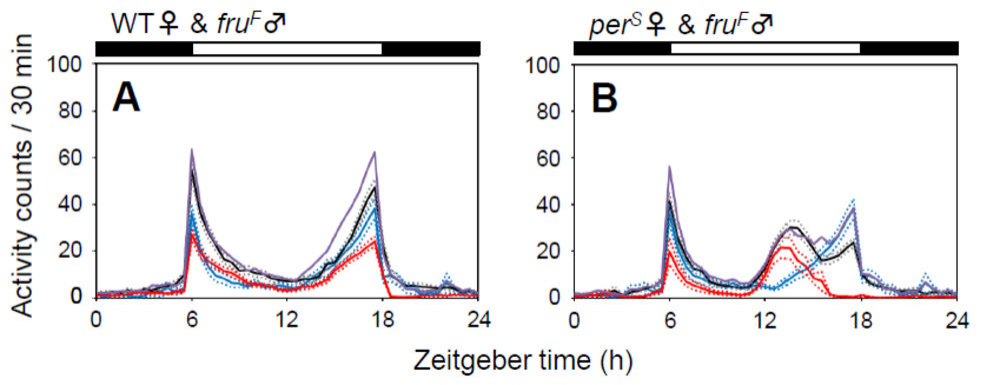
Pairing with *fru* ^*F*^ *Drosophila* mutants. (A) Single WT female flies paired with single *fru* ^*F*^ male flies (black line, n = 62; blue line, n = 15; red line, n = 61). (B) Single *per* ^*S*^ female flies paired with single *fru* ^*F*^ male flies (black line, n = 44; blue line, n = 15; red line, n = 16). The activity modulation induced by male-female interactions was suppressed in heterosexual couples that included *fru* ^*F*^ males. Activity of the two flies or single flies was recorded for 6 days in LD cycles and the average activity profiles were calculated from data of the last 5 days. For more detailed information, see Figure 1.

 The most prominent interaction between male and female flies is courtship behavior. The fruitless (fru) gene, which has sex-specific splicing forms, plays a very important role in male courtship behavior [14]. Female-specific *fru* ^*F*^ splicing mutants allow the production of only female-specific FRU protein, so that *fru* ^*F*^ males show a strongly suppressed courtship behavior toward female flies [18]. In *fru* ^*F*^ male-WT female pairs, no increase in night activity was observed (Figure 4A). Furthermore, in *fru* ^*F*^ male-*per* ^*S*^ female pairs, both the *per* ^*S*^ peak and the normally timed *fru* ^*F*^ peak were clearly visible in the pooled data (Figure 4B). Thus, the suppression of male courtship behavior abolishes activity modulations induced by heterosexual couplings. 

**Figure 5:**
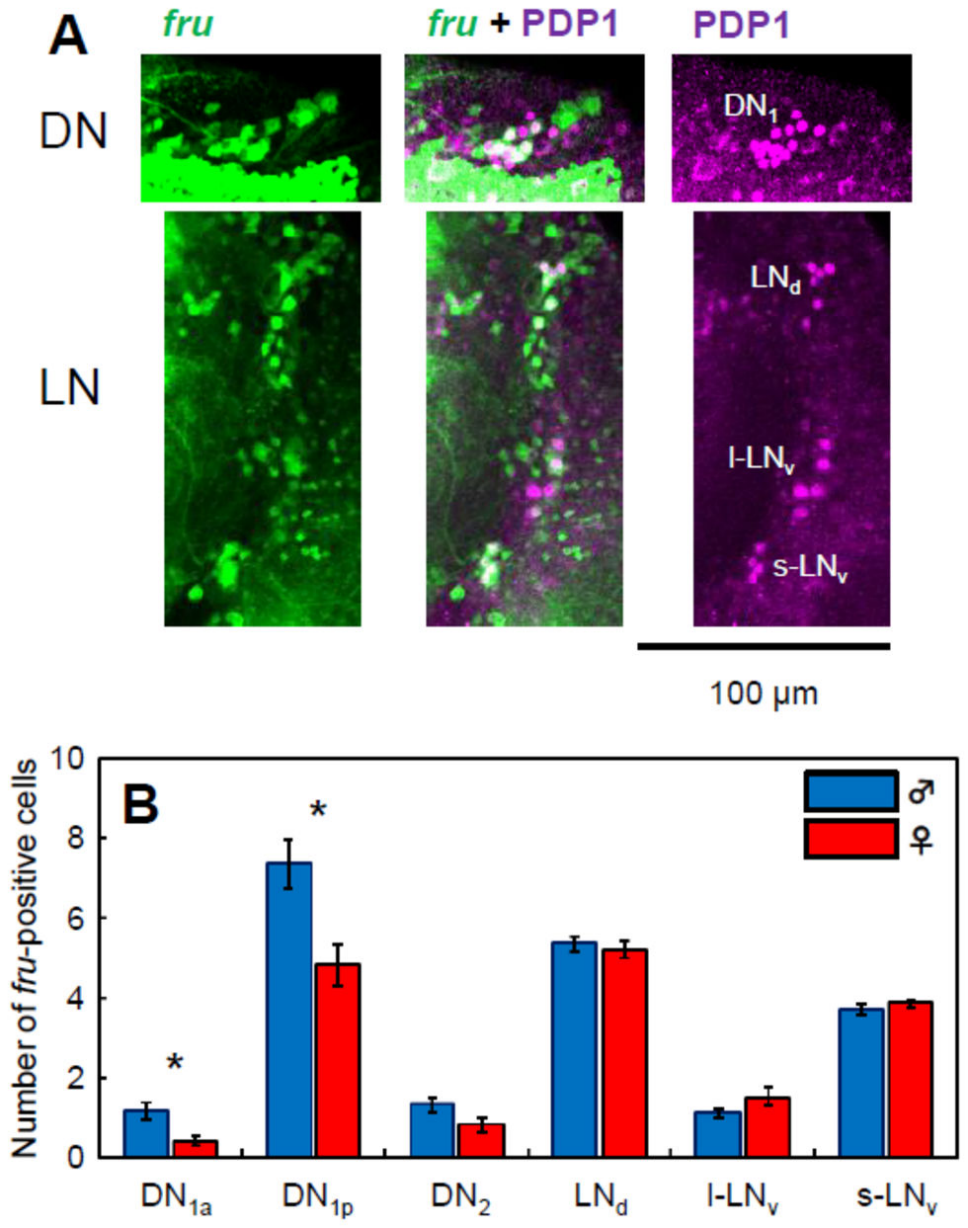
Fru expressions in the pacemaker neurons of the brain of *Drosophila*. (A) Double staining for *fru* expression visualized by GFP (green) and PDP1 expression by anti-PDP1 antibody (magenta) in the male brain. *fru* expressing cells are co-labeled by PDP1 staining in a subset of the pacemaker neurons. (B) The number of *fru*-positive pacemaker cells in the brains of males and the females. The data were obtained from 20 hemispheres of 10 brains for each sex. There is sexual dimorphism in the number of the *fru*-positive DN1 cells (*p < 0.05; Mann-Whitney U test followed by Bonferroni correction).

 A previous study showed that the *fru* gene is expressed in several pacemaker neurons of the s-LNv, LNd, DN1 groups [35]. We confirmed this using *fru-gal4/uas-GFP* flies and immunostaining with anti-PDP1 antibody. Our staining clearly demonstrated that of all s-LNv (including 5^th^ s-LNv) and LNd cells, only one I-LNV cell and 1~2 DN2 cells are *fru*-positive neurons (Figure 5). Interestingly, there is a sexual dimorphism in the number of *fru*-positive cells in the DN1 group: 2–3 more *fru*-positive DN1 cells are present in male brains than in female brains. The *fru-gal4* line that we used here is identical to the one used in the previous study [32]. This *gal4* line was created by a targeted insertion of the *gal4* gene into the *fru* locus and the reliable expression of *fru-gal4* has been proven in male and female brains [18].

### The molecular clock in DN1 pacemaker neurons is influenced by sexual interactions

 Finally, we investigated the effect of sexual interactions on the molecular clock in pacemaker neurons. Previous studies have revealed that the pooled activity rhythm in heterosexual couples is dependent on the male’s behavior [16,29]. In other words, the presence of females influences the males’ activity to alter the pooled activity of the two flies. Therefore, we speculated that the male’s clock may be synchronized by the female’s rhythms through courtship behavior. 

**Figure 6:**
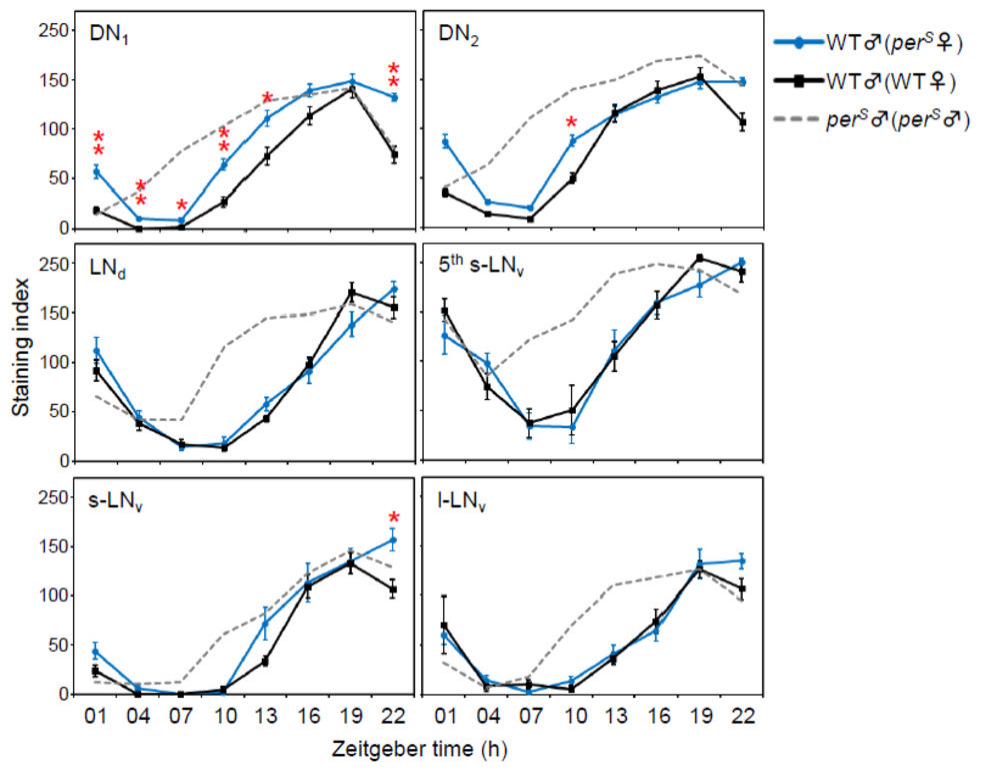
PDP1 oscillations in the pacemaker neurons of *Drosophila* during socio-sexual interactions. The molecular oscillations of PDP1 in each pacemaker neural group in WT male brains were measured by immunostaining. WT male flies were paired with either *per* ^*S*^ female flies or with WT female flies as a control group. For further comparison, the PDP1 rhythms in *per* ^*S*^ males paired with *per* ^*S*^ males are shown (gray dashed lines). **p < 0.01, *p < 0.05; t-test for normally distributed data and Mann-Whitney U test for data that was not normally distributed followed by Bonferroni correction. The PDP1 oscillations in the DN1 cells of WT male brains show an earlier increase in males paired with *per* ^*S*^ female flies.

A single pair of male and female flies was maintained in an acrylic tube, as in the behavior experiments, and only male flies from individual tubes in LD 12:12 were sampled at 3-h intervals. In the first experiment, WT males were paired with *per* ^*S*^ females. For the control condition, WT males were paired with WT females. If the male’s clock is synchronized by the presence of a single *per* ^*S*^ female, the molecular oscillations of the male’s molecular clock may shift toward *per* ^*S*^ oscillations. While most of the cell groups did not show this phase-shift, the PDP1 oscillations in the DN1 cells of WT males that were paired with *per* ^*S*^ females phase-shifted slightly, but significantly, toward those of *per* ^*S*^ males paired with *per* ^*S*^ males controls (Figure 6). Although the phase of the PDP1 peak did not differ between the two groups, the DN1 cells of WT males that were paired with *per* ^*S*^ females showed an earlier increase of PDP1 from ZT 10 compared with the controls. 

**Figure 7:**
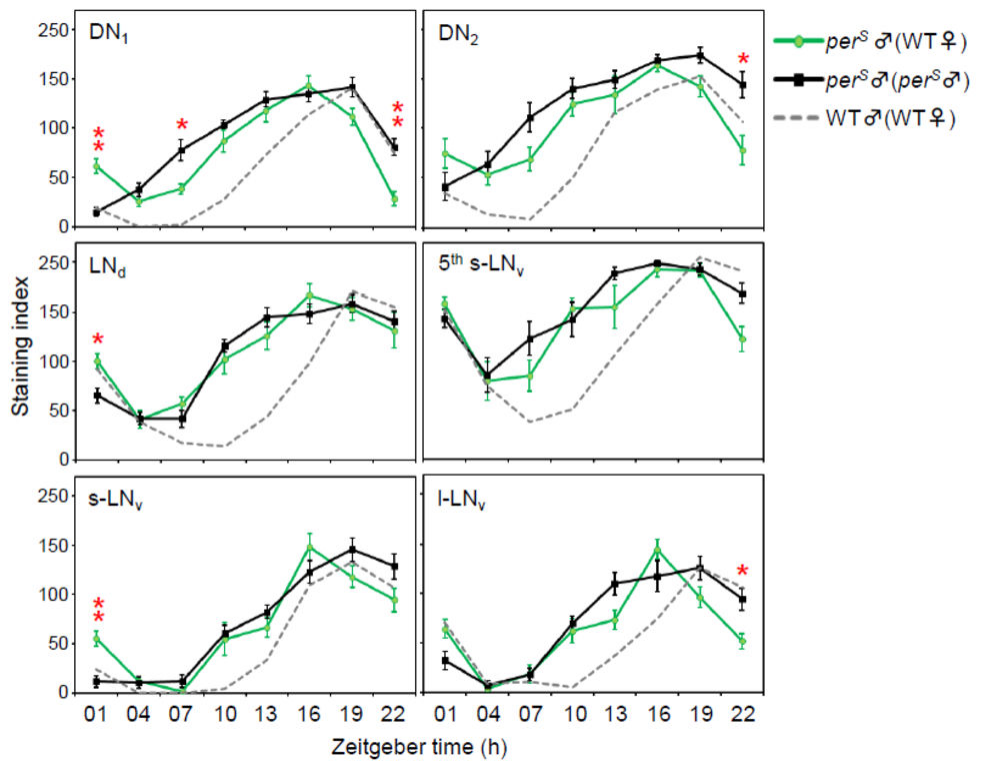
PDP1 oscillations in *per* ^*S*^ male-WT female *Drosophila* pairs. PDP1 oscillations in each pacemaker neural group were measured from perS male brains that were paired either with WT females or with perS males. The gray dashed lines indicate PDP1 rhythms in WT males that are derived from Figure 6. **p < 0.01, *p < 0.05; t-test for normally distributed data and Mann-Whitney U test for data that was not normally distributed followed by Bonferroni correction. Although the effect was weaker than in Figure 6, the increase of PDP1 in the DN1 cells was slower in perS males that were paired with WT females than in the control group.

 In the second experiment, *per* ^*S*^ males were paired with WT females. For a control group, *per* ^*S*^ male flies were paired with *per* ^*S*^ male flies to examine effect of the homosexual coupling by comparing with *per* ^*S*^ males-WT females couples. In general, the results were similar to those of the first experiment, as there was no clear difference between the two groups, except for the timing of PDP1 Increase in DN1 cells. This suggests that homosexual couplings of the same strains have no effect on the PDP1 oscillations. While the effect was weaker than in the first experiment, the increase of PDP1 in DN1 cells during the daytime was slightly slower than that in the control group (*per* ^*S*^ male-*per* ^*S*^ male) (Figure 7). Although other pacemaker neurons showed minor differences between the two groups, they were not consistent with the first experiment. These data indicate that male DN1 cells are influenced by the presence of a heterosexual partner. 

### Flies lacking functional clocks only in the DN1 cells

**Figure 8:**
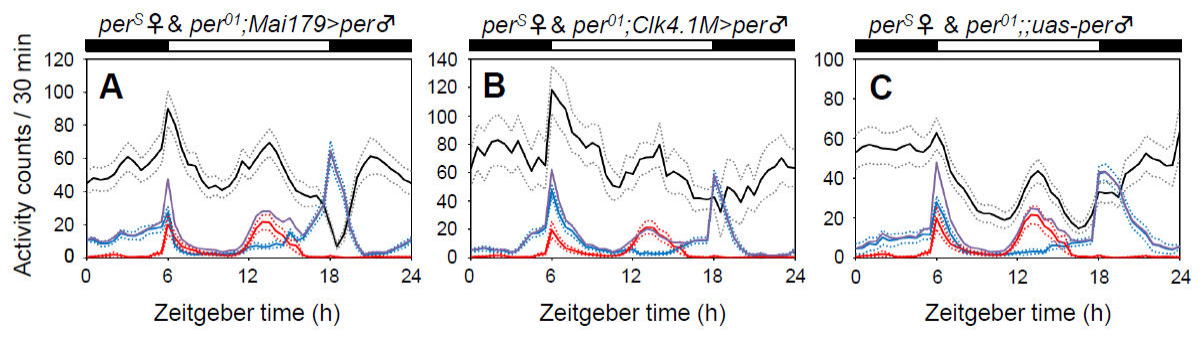
Effect of the presence/absence of the clock in the DN1 cells of *Drosophila*. per01; Mai179-gal4/+;uas-per/+ (per01; Mai179-gal4>per) flies were used to rescue the per gene in most of the LN groups, but not in the DN groups. In contrast, per01;;Clk4.1M-gal4/uas-per (per01;Clk4.1M-gal4>per) flies were used to rescue per in only a certain subset of the DN1 cells. (A) Pairs of perS female and per01; Mai179-gal4>per male flies (black line, n = 40; blue line, n = 21; red line, n = 16). (B) Pairs of perS female and per01;Clk4.1M-gal4>per male flies (black line, n = 44; blue line, n = 15; red line, n = 16). (C) Pairs of perS female and per01;; uas-per male flies (black line, n = 29; blue line, n = 31; red line, n = 16). Activity of the two flies or single flies was recorded for 6 days in LD cycles and the average activity profiles were calculated from data of the last 5 days. For more detailed information, see Figure 1.

 PDP1 cycling of DN1 cells in the male brains suggests that females may entrain the male’s DN1 cells, thereby the activity rhythms of female-male pairs are modulated. *Mai179-gal4* is expressed in all s-LNv cells and in a few l-LNv and 3 CRY-positive LNd cells, but not in DN1 cells [12,36,37]. In contrast, *Clk4.1M-gal4* is expressed only in the CRY-positive DN1p cells [21]. Crossing them with *per* ^01^;;*uas-per* strains, *per* expression can be rescued only in a subset of LN groups (*Mai179-gal4*) or only in a subset of DN1 cells (*Clk4.1M-gal4*), resulting in functional clocks in these cells [12,21]. Single male flies that have functional clocks in LN groups or a subset of DN1p groups exhibited quite normal activity rhythms with anticipatory activity increase before lights-on and lights-off, respectively (Figure 8). This is consistent with previous studies [12,21], and suggests that *per* expression was properly rescued in each cell group in this experiment. 

Males of these *per* rescue strains were paired with *per* ^*S*^ females to determine whether the presence or absence of the clock in the DN1 cells in males affected the pooled activity pattern. For the *Mai179-gal4 per* rescue flies, the pooled activity pattern was very similar to that for pairs of WT males and *per* ^*S*^ females: night activity increased relative to that of controls and there was a pronounced *per* ^*S*^ evening peak (Figure 8A). The *Clk4.1M-gal4 per* rescue flies also showed a similar activity pattern to that of WT male-*per* ^*S*^ female pairs (Figure 8B). There was no evident difference between *Mai179-gal4 per* rescue flies and *Clk4.1M-gal4 per* rescue flies paired with *per* ^*S*^ females, except for a strong reduction of evening activity in pairs of *Mai179-gal4 per* rescue males and *per* ^*S*^ females. Thus, the presence or absence of the functional clocks in DN1 cells is not very important for the heterosexual-induced activity modulation.

In a control experiment, pairs of *per* ^*S*^ females and *per* ^01^;;*uas-per* males display a clear *per* ^*S*^ evening peak at the middle of the day as well as high night activity (Figure 8C), which is again similar to what WT male-*per* ^*S*^ female pairs show. This suggests that the circadian clock in males does not play an important role in the pooled activity pattern in the heterosexual couples. 

## Discussion

 Here, we focused on sexual interactions as a potential time cue for the circadian clock. *Drosophila* does not exhibit sophisticated social behaviors like those of other social insects, such as bees, ants, or aphids. Therefore, it could be argued that *Drosophila* may not be well suited to the study of this subject. This would be true if the focus of the investigation was the role of such “sophisticated” social interactions in the circadian clock. However, the lack of sophisticated social behaviors is unlikely to affect the relationship between primitive social behaviors and the circadian clock. Mating behavior—males and females interacting socially to produce offspring in a species-specific manner—is fundamental in most animals. In *Drosophila*, the timing of mating time is controlled by the circadian clock [16,38,39], which provides a temporally efficient means of meeting with mating partners and eventually results in conserving energy. However, it is unclear whether circadian rhythms can adapt to the rhythms of partners to improve mating success. The results of this study and those of previous studies suggest that, in the presence of female(s), the activity rhythms of male flies differs from their activity rhythms in the absence of females [29]. However, unless the oscillation of the molecular clock is similarly affected, this does not mean that the male-female interactions can serve as a zeitgeber. 

### Courtship behavior and circadian activity rhythms

 The enhancement of night activity induced by heterosexual coupling observed in the present study fits well with a phenotype described in a previous study, in which close-proximity between male and female flies was measured in the context of circadian rhythms [16]. In the earlier study, video-recording-based automatic analyses revealed that heterosexual couples placed in a 35-mm-diameter Petri dish physically interact at night and in the morning. This suggests that there is a higher frequency of the courtship activity during the night and morning than at other times. Two other studies made direct observations of the timing of fly mating [38,39] and clearly showed that many mating behaviors occur during the subjective night. Our behavioral experiments involving *fru* ^*F*^ mutant males also suggest that the high night activity in WT heterosexual couples is due to mating behavior. Male flies may chase rejecting females during the night. 

Male flies lacking the third antennal segment or a specific olfactory receptor, *Or83b*, have a severely reduced number of close-proximity encounters with females during the night and morning [16]. Furthermore, the *Or47b* receptor in male flies is required for the pooled night and day activity of heterosexual couples to increase [29]. Thus, it is likely that, at night, the olfactory stimulus to males induces courtship behaviors and the pursuit of females, which results in an increase of night activity. The observed increase in night activity is not mediated by the circadian clock, as the same behavior was also seen in *per* ^01^ mutants (Figure 1). 

### The phase of female evening activity leads male activity

 When a potential partner has a different active time, the other may adapt to the activity pattern of the potential mate. Interestingly, in pairs of WT and *per* ^*S*^ flies, the activity of the pairs increased during the female’s evening peak, whereas activity tended to be reduced during the male’s evening phase (Figure 2). Since female flies are generally more active than male flies [30], we assume that a running female stimulates a paired male, promoting courtship behaviors and eventually provoking high levels of activity of the male fly during courtship. In addition, this high activity may cause sleep rebound, thereby reducing activity during periods in which the male would normally be active.

 We expected that this plastic behavioral change would be due to that the male’s clock synchronizes to the female’s rhythm, but it is not. The pooled activity pattern of *per* ^*S*^ female-*per* ^*01*^ male pairs does not differ so much from that of *per* ^*S*^ female-WT male pairs, indicating that the male’s clock does not contribute to the activity rhythm of heterosexual couples (Figure 8C). When two flies were separated, both flies immediately reverted to single-fly activity patterns without any sign of circadian entrainment (Figure 3). These results suggest that the circadian clock is not synchronized by sexual-interactions. The same conclusion was also reached by Fujii and co-workers [16], although they only examined the behaviors of WT flies after couples were separated. In grouped male flies, individual activity rhythms have a synchronized phase [5,40]. Importantly, the effect of the phase synchronization decreases with group size. This may explain why we did not observe any such synchronization in heterosexual pairings in the present study. Lone and Sharma (2011) kept 30 flies (15 males + 15 females) in a vial for some days, and the activity rhythms of individual flies were recorded [15]. The evening peak was reduced and the free-running period was lengthened in male flies that experienced socio-sexual interactions. The lengthened free-running period clearly suggests an effect on the circadian clock. The coupling of a single male fly with a single female fly in a small tube may be an inappropriate experimental condition and is not a natural situation for flies. 

### DN1 cells may sense social time cues

Despite our behavioral data that does not support the potential of social interactions as a zeitgeber, we performed experiments to determine whether the males in WT and *per* ^*S*^ heterosexual couples flies were synchronized at the neural pacemaker level by the females. Visible and consistent effects in two independent experiments were seen only in the DN1 group (Figures 6, 7), but the effect was not very strong. 

The role of the DN1 group is not well understood. The CRY-negative DN1 neurons are sensitive to temperature changes [22,41], implying that the cells in this group may be important for temperature entrainment. DN1 neurons are also regarded as the oscillators involved in the evening activity of *Drosophila* [13,42]. DN1 cells also play a role in activity rhythms under constant light [43,44]. These reports do not provide a coherent conclusion that explains the functions of the DN1 pacemaker neurons. This may be because DN1 cells are a heterogeneous group of cells [36,45]. The present study also revealed that, in addition to expression in LNs, the *fru* gene is expressed in a certain subset of DN1 cells, and the number of the *fru*-positive DN1 neurons differs between the sexes (Figure 5). The weak effect of sexual interactions in DN1 cells could be due to the heterogeneity of the group. A subset of the DN1 cells (such as the *fru*-positive DN1 cells) may respond to social cues, whereas others may not. 

Using a *Drosophila* genetic tool, the GAL4/UAS system, two previous studies manipulated pacemaker neurons to investigate which neurons are involved in the circadian rhythms of close-proximity activity [35,46]. Their consistent finding was that the DN1 evening pacemaker neurons are important for normal close-proximity rhythms. Taken together, we hypothesize that DN1 cells may play a role in a circadian center for socio-sexual interactions that senses the social time cue and also provides temporal information for courtship behaviors. This hypothesis needs further study in order to be confirmed. 

In summary, the present study provides a good base for understanding the effect of socio-sexual interactions on pacemaker neurons. The results suggest that, in heterosexual couples, the male DN1 cells are synchronized to the female rhythms. In future studies, the circadian neural network responsible for social synchronization will be investigated in grouped flies. 
